# “Disruptive behavior” in the operating room: A prospective observational study of triggers and effects of tense communication episodes in surgical teams

**DOI:** 10.1371/journal.pone.0226437

**Published:** 2019-12-12

**Authors:** Sandra Keller, Franziska Tschan, Norbert K. Semmer, Eliane Timm-Holzer, Jasmin Zimmermann, Daniel Candinas, Nicolas Demartines, Martin Hübner, Guido Beldi

**Affiliations:** 1 Institute of Work and Organizational Psychology, University of Neuchâtel, Neuchâtel, Switzerland; 2 Virginia Tech, Blacksburg, VA, United States of America; 3 Institute of Work Psychology, University of Bern, Bern, Switzerland; 4 Department of Visceral Surgery and Medicine, University Hospital of Bern, Bern, Switzerland; 5 Department of Visceral Surgery, Lausanne University Hospital CHUV, Lausanne, Switzerland; Louisiana State University, UNITED STATES

## Abstract

**Background:**

Tense communication and disruptive behaviors during surgery have often been attributed to surgeons’ personality or hierarchies, while situational triggers for tense communication were neglected. Goals of this study were to assess situational triggers of tense communication in the operating room and to assess its impact on collaboration quality within the surgical team.

**Methods and findings:**

The prospective observational study was performed in two university hospitals in Europe. Trained external observers assessed communication in 137 elective abdominal operations led by 30 different main surgeons. Objective observations were related to perceived collaboration quality by all members of the surgical team. A total of 340 tense communication episodes were observed (= 0.57 per hour); mean tensions in surgeries with tensions was 1.21 per hour. Individual surgeons accounted for 24% of the variation in tensions, while situational aspects accounted for 76% of variation. A total of 72% of tensions were triggered by coordination problems; 21.2% by task-related problems and 9.1% by other issues. More tensions were related to lower perceived teamwork quality for all team members except main surgeons. Coordination-triggered tensions significantly lowered teamwork quality for second surgeons, scrub technicians and circulators.

**Conclusions:**

Although individual surgeons differ in their tense communication, situational aspects during the operation had a much more important influence on the occurrence of tensions, mostly triggered by coordination problems. Because tensions negatively impact team collaboration, surgical teams may profit from improving collaboration, for instance through training, or through reflexivity.

## Introduction

The picture of the cursing surgeon that throws instruments across the operating room (OR) is still present in many minds. Although such extreme events are nowadays rare [1], tense communication has not disappeared from modern ORs [2,3]. Several studies observed between one and four moments of tense communication per surgical procedure [4,5]; Jones and colleagues [1] reported that 2.8% of all communications in the OR were tense.

Expressed tensions can be described as communications emitted in a negative affective tone; they include the expression of dissatisfaction and incivilities of different intensity. Tensions thus range from subtle negative behaviors to overt aggressive communication such as yelling [6] or using condescending language [7]. A characteristic of these behaviors is that they do not necessarily imply an intent to harm: the instigator may even be unaware of potential harm caused [8]. Note that this definition is broader than definitions that limit disruptive behavior as socially and professionally inappropriate conduct [9]. These behaviors can, however, be characterized as signs of impaired “relational coordination” [10].

### Triggers of tensions

Uncivil behaviors are by no means restricted to surgeons, or to supervisors in general [11]. Nevertheless, attending surgeons are more likely than other professional groups to express tensions in the OR [12–15]. A common explanation regards personality or personal communication style of the surgeons as the main reason for tense communication [15], corresponding with studies in other medical teams, where physician personality is one of the most frequently mentioned reason for tensions [2,9,16]. Such attributions perceive tensions to be primarily caused by specific individuals, often known as “bad apples” [9,15] or bullies [17]. Other studies suggest that anxiety, depression, aggressiveness or prior victimization of surgeons increase the likelihood of dysfunctional behavior [9]. The prominent role in the OR hierarchy [7] and the surgeon’s status in the organization potentially contribute to the persistence of these behaviors as well [9,18,19].

However, explanations that focus on personal predispositions are likely to underestimate contextual factors that trigger tense episodes. Stress, as well as production and time-pressure may further increase tense communication [9,10,14]. Many tensions are indeed related to overrunning or changing scheduled surgeries and to availability of theatre time, staff or equipment [5,20–22]. During surgical procedures, unexpected intra-operative complications, but also divergent opinions related to safety have been found to trigger tensions [15], as have task-execution and collaboration problems [4,14]. Thus, focusing solely on personality and person aspects when explaining tensions in the OR is not sufficient, and research on tensions in the OR requires observational studies looking beyond personal aspects of team members as reasons for tensions [7] and impaired relational coordination in general [10].

The primary goal of this prospective observational study was to assess tense communication episodes in the OR, with a focus on its triggers, and to distinguish between personal and situational influences on the occurrence of tense communication in the OR.

### Effects of tensions

The second goal of this study focusses on the potentially negative consequences of tense communication for the quality of teamwork in the OR [23].

Tense communication can elicit negative emotions, which may decrease attentional resources [24], shift the attention to the perpetrator [25], and thereby hamper individual performance in the OR [26]. Tensions have negative effects for the OR team [9]. After tense episodes, team members minimize communication [27,28] and prosocial behavior [28]; and even mild display of negative emotions can impair speaking up [29,30]. In addition, tensions may impair team learning [31]. Thus, tensions most likely have a negative impact on the quality of collaboration within surgical teams.

However, the negative effects of tensions depend on the situation that triggered the tension [32], as people take the context into account when interpreting social communication [33]. For example, if a surgeon is faced with a very difficult aspect of the surgical task and complains with high tension about an apparatus that is not functioning, team members may attribute the tension to the surgeon’s stress. They thus may experience such a tension as less threatening, as compared to a situation in which the surgeon reacts angrily because he or she is not satisfied with the cooperation, for example by how instruments are handed. This implies that the negative effect of tensions on collaboration quality may be different if the tense communication is triggered by collaboration problems (where someone could be blamed), as opposed to problems due to the difficulty of the task at hand or to malfunctions that no one present is responsible for [32].

In general, more conflict or tensions in teams are related to lower performance, but also to lower satisfaction of team members [34–36]. In surgical teams, tensions potentially impair collaboration quality, and an association between tensions and worse patient outcomes or medical errors [2,7,9] is assumed [26].

Second goal of this study was to assess effects of observed tensions during surgeries on the perceived collaboration quality within the surgical team.

## Materials and methods

This prospective observational study was part of a larger study that received ethical approval by the Institutional Review Board of the canton of Bern (KEK-BE #161/2014; leading ethics committee) and the canton of Vaud (CER-VD #2016–00991). No patient data was used in this study. Participating surgical team members were extensively informed at dedicated presentations and received written information about the study. Consent of surgical team members to be observed was assured by an opt-out procedure–participants could at any time refuse the presence of the observer in the operating room. Oral consent was obtained from team members each time before filling in the questionnaire. Participants expressed their consent by accepting to answer to the questionnaire. No written record of participant name was collected.

In two university hospitals, 137 abdominal operations, led by 30 different main surgeons, were observed. Inclusion criteria were availability of the observers and elective surgical procedure. All team members in the OR were aware of the presence of the observers and were informed that communication and distractions were observed.

### Observing and coding tense communication in the OR

The surgical procedures were observed by trained observers (industrial psychologists) using a validated observational system for assessing communication and distractions in the OR [37]. Interobserver reliability was assessed by Cohen’s kappa, separately for each observational code; all Cohens kappa were above 0.70. The observers were present in the OR, sitting about 150 cm away from the operating table. Tablet computers were used for coding, and the coding software [38] automatically timestamped observed events. Observation time was between the WHO timeout checklist (immediately prior to incision) and the last stitch.

A tense communication was coded if a verbal message of a member of the surgical team was expressed in an annoyed or angry tone [4,39]. This definition is broad and ranges from the expression of mild annoyance not targeted at other team members to open insults. If a tension was observed, observers used a comment function on the tablet to describe the content of the communication and the circumstances. These open descriptions were content-coded by two independent coders (SK and FT) [40]; disagreements for this coding were resolved in discussion.

#### Source and target of tensions

Coders noted the person who initiated a tense communication and who, if anyone, was the target towards whom the tense communication was directed. Codes for initiators referred to all team members, that is, main surgeon, second surgeon, resident, scrub technician, anesthetist, circulator, plus non-team members (e.g., external visitors). For the tensions that were aimed at specific team members (targets of tension), the same codes were used with the exception that surgeons (main, second or resident) were not distinguished; furthermore, a code for tensions targeted at the whole team (room), and a code for undirected tensions were added.

#### Coding triggers of tensions

Based on the observers’ summaries of what was communicated during a tension episode, triggers of tensions were coded. Note that more than one trigger was possible for some tensions. Code development was based on previous research [39,41,42] on the one hand, and inductively derived from the material on the other hand. Our examples refer mainly to surgeons because the surgeons initiated most of the tense communication (see results section). *Task-related tension trigger* was coded if tensions were triggered by disagreements about the procedure, by problems related to the procedure (e.g. the surgeon expresses anger about the complicated anatomy of the patient), by the work organization (e.g. a surgeon complains about shift changes in the anesthesia team), by delays that cannot be attributed to a specific person (e.g. a device is not working correctly) and by too much noise or distractions (e.g. a surgeon complains about a high noise level in the OR. *Coordination-related tension-trigger* was coded if the trigger was related to collaboration difficulties (e.g. a surgeon demands that the scrub technician hands the instruments faster; a surgeon expresses anger about being handed the wrong type of thread), triggered by an inappropriate or inept act of another person (e.g. the surgeon shouts at a resident who obstructs her view; a nurse tells a resident in an angry tone that he *really* needs to find that clamp), or triggered by a perceived lack of competence of a team member (e.g. the surgeon angrily tells the scrub technician that she should know by now that he always uses a specific type of thread). *Other tension triggers* was coded for tensions without an obvious relationship to the current case (e.g. the surgeon blames a resident for discharging another patient without full information), and interpersonal tensions that do not have a clear trigger but indicate a tense relationship between two individuals (e.g. a scrub technician and a surgeon have difficulties collaborating and show signals of interpersonal disaffection).

#### Assessing teamwork quality

Before leaving the OR, each member of the surgical team (all surgeons, anesthetists, scrub technicians, and circulators) were asked by the observers to individually fill out a short questionnaire related to the procedure. Up to three questions assessed the perceived quality of teamwork within, or with, the surgical team. Main and second surgeons answered tree questions, assessing collaboration quality with the other surgeons, the scrub technician and anesthetists separately. An example is “How was the collaboration among the surgeons”. For the surgeons, the three questions were combined into a scale. Cronbach’s alpha for this scale was 0.69 for main surgeons, and 0.82 for second surgeons, indicating acceptable to high internal consistency. For scrub technicians, the question was “How was the collaboration within the surgical team”; and for anesthetists and circulators it read “How was the collaboration with the surgical team”. Answers were on a seven-point Likert scale from 1 (very bad) to 7 (very good). For the perception of teamwork quality by the team as a whole, values of the individual members were averaged. As different team members may have very different perspectives, this index cannot be expected to be unidimensional, and indeed, Cronbach’s alpha was 0.50. Even though agreement about teamwork quality among surgical team members is not very high, an average value nevertheless can be regarded as an overall indicator.

#### Outcomes

Primary outcome of this study was triggers of tense communication episodes during surgical procedures. Triggers included task-related, coordination-related, and other triggers. Secondary outcome was teamwork quality as a function of tense communication, as well as the relationship of tensions triggered by task versus coordination issues with teamwork quality.

#### Statistical analyses

We used descriptive statistics (counts, per cent, mean, standard deviations) for descriptive results. We used t-test to assess differences between hospitals; χ^2^ test to assess differences between surgeons with regard to absence or presence of tense communications, and analysis of variance to assess differences in the mean number of tensions across different surgeons.

To assess effects of tensions on perceived collaboration quality, we used random intercept multilevel multiple regression analyses. Multilevel regression is appropriate because surgeons performed several surgeries. The main surgeons therefore were treated as predictor on the second (higher) level; all other variables, notably the situational triggers, were predictors on level 1 (surgery). Results were adjusted for hospital and for duration of the surgery. Multilevel regression was also used to calculate the variance partitioning value as the percentage of variance associated with individual surgeons or with the context (surgery) when estimating the mean number of tensions. IBM SPSS Statistics for Windows, Version 25.0, released 2017 in Armonk, NY by IBM, was used for data analyses; P <0.05 was considered significant.

## Results

### Descriptive results

A total of 137 surgeries were observed in the department of general surgery of two European University hospitals (86 in hospital 1, 51 in hospital 2, Table 1). Mean duration between incision and closure was 3.67h (SD = 2.21), with no significant differences between the two hospitals (M_hospital 1_ = 3.74h, SD = 2.43; M_hospital 2_ = 3.55h, SD = 1.89; t = 0.491, P = 0.624). The surgeries were led by 30 different main surgeons (Hospital 1: 17 Hospital 2: 13); per surgeon, between 1 and 17 surgeries were observed.

**Table 1:** Surgical procedures observed.

Surgical procedure	Hospital 1	Hospital 2	Percent
Lower gastrointestinal tract	16	12	20.4%
Upper gastrointestinal tract	6	9	10.9%
Liver	20	6	19.0%
Pancreas	16	10	19.0%
Bariatric (bypass/gastric sleeve)	6	4	7.3%
Kidney transplant	10	0	7.3%
Hernia	4	2	4.4%
Other	8	8	11.7%
Total	86	51	100.0%

Across all surgeries, a total of 340 tense communication episodes were observed, which amounts to 2.48 (SD = 5.19) tensions per surgery or 0.57 tensions (SD = 1.02) per hour of surgery. Note that 72 surgeries (52.6%) were tension-free. Mean number of tensions for surgeries with at least one observed tension was 5.23 (SD = 6.53), or 1.21 (SD = 1.19) per hour of surgery.

**Fig 1:**
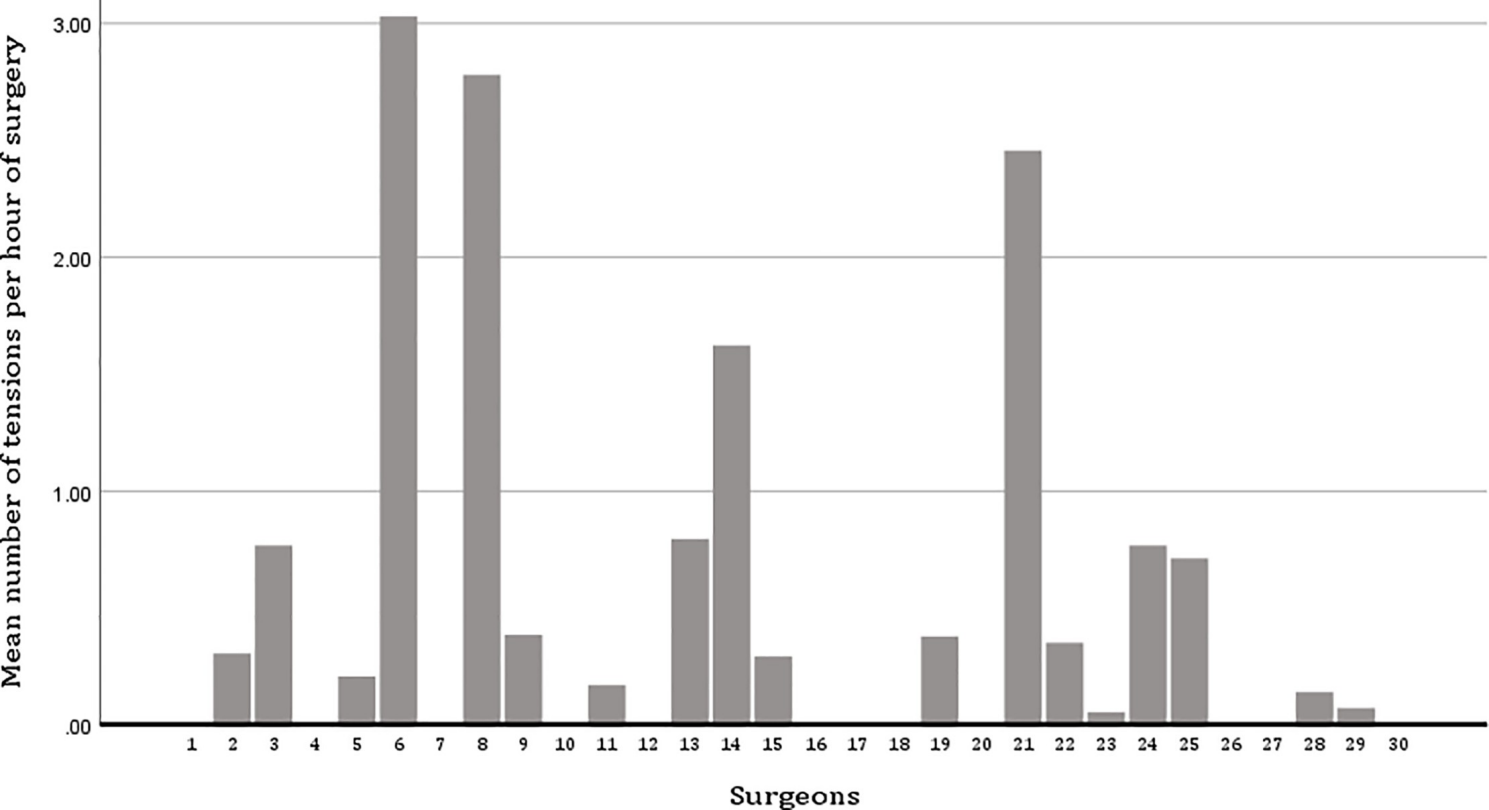
Mean tensions per hour across all 30 participating surgeons.

No tense communication was observed in the operations of 12 main surgeons; in operations of the other 18 main surgeons, at least one tense communication was observed across all operations of these surgeons (Chi2 (df = 29) = 67.399, P<0.001); the number of tensions per hour varied significantly between surgeons (F(1,29) = 5.487, P<0.001; adjusted for hospital) (Fig 1). Multilevel analyses show that individual surgeons explain 24.01% of the variation in number of tensions (variance partition coefficient derived from multilevel regression), and the situational context explains 75.99% of the variation.

Almost all tensions were initiated by surgeons (main and second; 97.4%; Table 2); main targets of tensions were (other) surgeons and scrub technicians (Table 3).

**Table 2:** Initiators of tensions.

Initiator of tension	Frequency	Percent
Main surgeon	306	90.0%
Second surgeon	24	7.1%
Resident	1	0.3%
Anesthetist	1	0.3%
Scrub technician	5	1.5%
Circulator	2	0.6%
Unclear	1	0.3%

**Table 3:** Targets of tensions.

Targets of tensions	Frequency	Percent
Surgeon (main, second, resident)	109	32.1%
Scrub technician	95	27.9%
Circulators	31	9.1%
Anesthetist	25	7.4%
Non team members	11	3.2%
All, the room	5	1.5%
Unclear	69	20.3%

N = 340 tensions–as a tension can have several targets, thus the total sum of percent is greater than 100.

### Triggers of tensions

Task-related triggers for tensions were less frequent (21.3%) than tensions triggered by coordination problems (72.4%) (Table 4).

**Table 4:** Triggers of tensions.

Triggers of tensions		n	percent	n and percent per category
Task-related triggers				72 (21.2%)
	Disagreement about the procedure	0	0%	
	Problems related to the procedure	20	5.9%	
	Work organization	2	0.6%	
	Delays	24	7.1%	
	Noise or distractors	27	7.9%	
Coordination-related triggers				246 (72.4%)
	Collaboration difficulties	183	53.8%	
	Inept actions of others	122	35.9%	
	Perceived lack of competence	28	8.2%	
Other triggers				31 (9.1%)
	Not related to current case	11	3.2%	
	Interpersonal dislike	4	1.2%	
	Not codable	16	4.7%	

N = 340 tensions–as a tension can have multiple triggers, the total sum of percent is greater than 100

### Effects of tensions on teamwork quality

In general, quality of teamwork was rated as high by all members of the surgical team, with means between 5.58 and 6.14 on a 7-point scale (Table 5). Main surgeons perceived significantly lower teamwork quality than second surgeons; anesthetists perceived significantly lower teamwork quality than all other professions; no other differences between professions were significant.

**Table 5:** Tensions per hour and perceived teamwork quality.

		Unadjusted results				Adjusted results			
Teamwork quality		tensions, p hour				tensions, p hour			
	M (SD)	B	95% CI low	95% CI high	P	B	95% CI low	95% CI high	P
Main surgeon	5.94 (0.87)	-0.165	-0.323	-0.008	0.040	-0.216	-0.378	-0.055	0.009
Second surgeon	6.02 (0.80)	-0.196	-0.343	-0.048	0.010	-0.253	-0.404	-0.102	0.001
Anesthetists	5.58 (0.98)	-0.152	-0.326	0.022	0.085	-0.131	-0.323	0.062	0.181
Scrub technicians	6.02 (1.26)	-0.525	-0.728	-0.321	0.000	-0.479	-0.706	-0.253	0.000
Circulator	6.14(0.89)	-0.314	-0.469	-0.159	0.000	-0.241	-0.407	-0.075	0.005

Dependent variables are perceived teamwork quality by the different professions (range from 1 to 7). Based on multilevel regressions, adjusted for hospital, duration of surgery; N Level 1: 137 surgeries, N Level 2: 30 surgeons.

**Fig 2:**
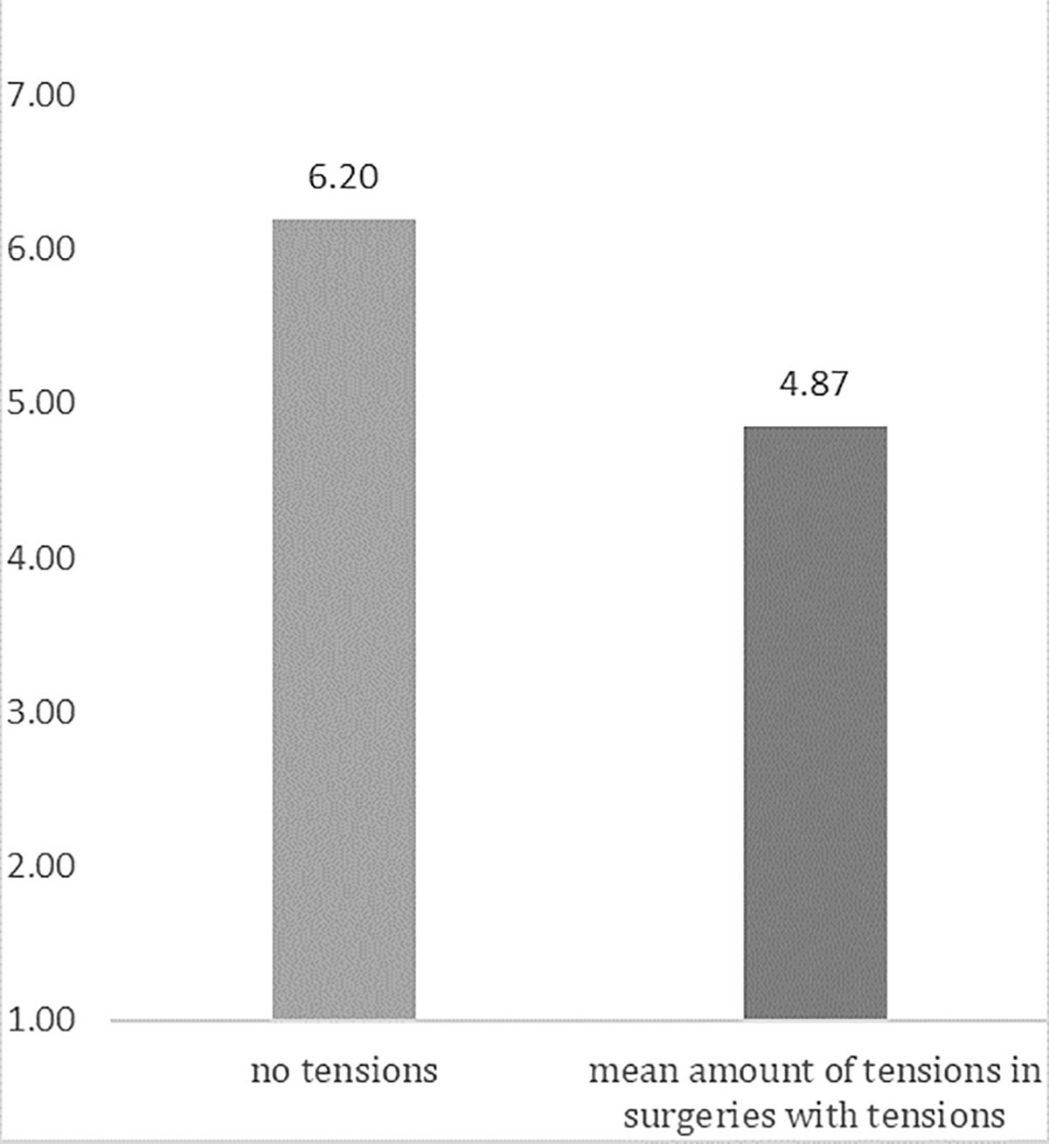
Illustration of perceived teamwork quality by the different professions for surgeries with no tensions as compared to surgeries with the mean level of tensions (in surgeries with tensions).

Separate multilevel regression analyses for each profession showed that more observed tensions were significantly related to lower perceived teamwork quality for all team members except the anesthetists. This effect persisted when adjusting for hospital and duration of surgery (Table 5). If a mean score of teamwork quality across the whole team is calculated, each additional tension per hour was related to a decrease of about a quarter point [B = -0.256 (95% CI = -0.362 to -0.153)] in the teamwork quality index (adjusted for duration of surgery and hospital). Fig 2 illustrates this effect, comparing the estimated level of teamwork quality for surgeries without any tensions and the estimated level of teamwork quality for the mean level of tensions in surgeries containing tense behaviors.

**Fig 3:**
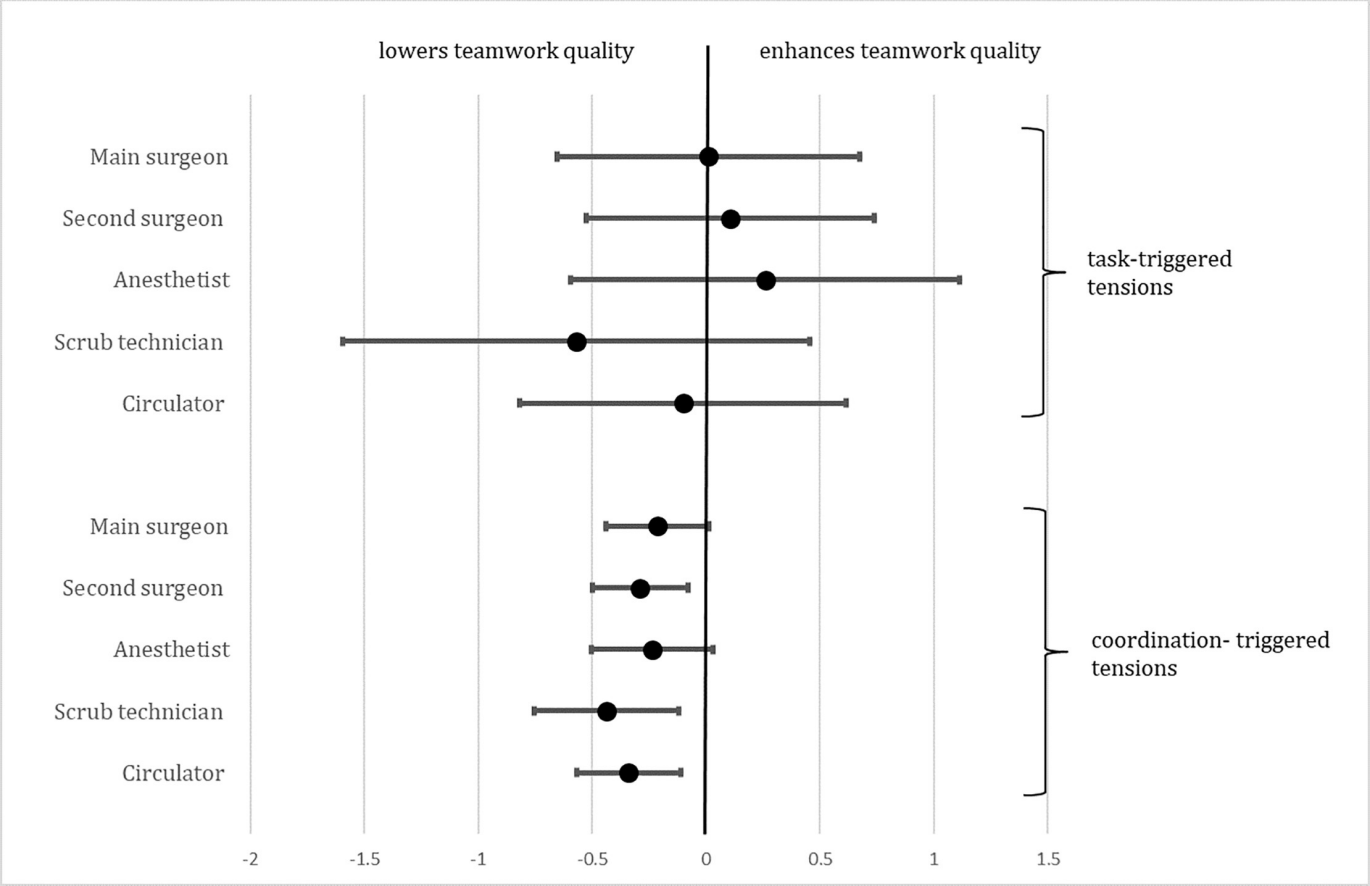
Illustration of effect of task-triggered and coordination-triggered tensions on teamwork quality for surgeons, anesthetists and scrub nurses.

Collaboration-triggered tensions were related to lower teamwork quality for second surgeons, scrub technicians and circulators, but not for main surgeons and anesthetists. Task-triggered tensions were not significantly related to teamwork quality for any professional group (Table 6 and Fig 3).

**Table 6:** Effects of task- and coordination-triggered tensions on quality of teamwork within the team.

	task-triggered tensions, ph				coordination-triggered tensions, ph			
	B	95% CI low	95% CI high	P	B	95% CI low	95% CI high	P
Main surgeon	0.010	-0.654	0.674	0.976	-0.213	-0.438	0.013	0.065
Second surgeon	0.104	-0.527	0.736	0.744	-0.290	-0.501	-0.079	0.008
Anesthetists	0.259	-0.595	1.113	0.550	-0.237	-0.502	0.029	0.080
Scrub technicians	-0.571	-1.596	0.455	0.273	-0.438	-0.754	-0.122	0.007
Circulators	-0.100	-0.817	0.616	0.782	-0.340	-0.569	-0.112	0.004

Dependent variable is teamwork quality as perceived by the different professions. Based on multilevel regressions; types of triggers are adjusted for each other and for other triggers, as well as for hospital and for duration of surgery; N Level 1: 137 operations, N Level 2: 30 surgeons.

## Discussion

In this study, 340 tense communications in 137 surgical procedures were observed. Across all surgeries, roughly one tension was observed every 1 hour and 45 minutes. Most of the tense communications were initiated by the main surgeon and most were targeted at other surgeons or at scrub technicians. Most tensions were triggered by coordination problems; task-related problems as triggers were much less frequent, and triggers related to interpersonal conflicts or disagreements were very rare.

As found in other studies [1,4] tension density was not high. However, about half of the observed surgeries contained tensions; this percentage is relatively high compared to other studies that found tense communication in only about a third of procedures [1].

### Surgeon personality or situational triggers?

Almost all tense communications were initiated by (main) surgeons and targeted at the closest collaboration partners at the surgical table–other surgeons and scrub technicians. More than 70% of tensions were triggered by coordination problems, such as collaboration difficulties, inept actions of others and perceived lack of competences; task-related issues were the second most frequent trigger. Note that tensions triggered by disagreement about the procedure were never observed, and tensions related to interpersonal dislike [42] were extremely rare.

At first sight, the finding that surgeons initiated most of the tensions, and that there were differences between surgeons regarding the occurrence of tense behaviors, corroborates attributing tensions in the OR to the “difficult surgeon personality” [2,9,16] and to the fact that the high status allows surgeons to vent their negative emotions [1,7,9]. It is undeniable that high-status team members have less trouble speaking up and can vent their negative emotions more easily [43,44]. Communicating one’s (negative) emotions is influenced by general, but also local social norms [45], and these may “allow” surgeons to show negative emotions more easily, whereas other team members have to suppress their display. However, in explaining the number of tensions, the analyses show that the context (surgery) was about three times as important than individual surgeons (24% vs 76% of variance explained by surgeons vs surgery, respectively).

Thus, when explaining the higher number of tensions emitted by surgeons, more than personality must be considered [15], and when surgical team members attribute disruptive surgeon behavior primarily to personality (and even assume a surgeon specific-personality), because this profession “attracts and creates individuals with particular personality traits” ([15], p. 390), they may well be biased and underestimate situational factors. This bias reflects the so-called fundamental attribution error, which denotes the general tendency to attribute the actions of others to stable characteristics of the person while underestimating situational influences [46]. The fundamental attribution error is enhanced if people judge negative actions, such as incivility, by members of another social group [47,48]. Indeed, an experimental study including video-based scenarios found that nurses, surgeons and anesthesiologist perceived the other professions to be more responsible for creating tensions—while watching the same video clips [49].

In this study, the triggers were content coded for each tension observed. The analysis of these triggers shows that tense communication occurred if the progress of the surgery was threatened by task or coordination difficulties. The main surgeon has several roles during surgery. Not only is the main surgeon the leader in the OR in terms of status [50,51], but at the same time, he or she performs most, and especially the most difficult, parts of the surgery proper. Furthermore, the task itself is “hierarchical” in character, as the actions of the surgeon are at its center, and actions of other members should enable and facilitate task execution by the surgeon, who, in the end, bears responsibility for the surgery.

Surgery is characterized by tasks that require high concentration on manual aspects, often in very tight and smooth cooperation with assisting surgeons and scrub technicians [52]. In this cooperation, time is critical, and timeframes for good cooperation may well include fractions of seconds. For example, handing instruments with even minimal delay can disturb the smooth process [52]; a similar argument can be made for the precision of movements. Problems in tight cooperation can obstruct goals or interrupt the workflow, may elicit anger and tense behavior, and may lower performance [53]. In this sense, tensions may serve as an indicator of coordination problems [54]. Indeed, in a study by Tørring et al. [55], actions such as nurses delivering an instrument simply based on their anticipation of what would be needed are cited as examples of good relational coordination.

### Tensions reduce teamwork quality

More tensions were found to be related to perceptions of lower teamwork quality for all members of the surgical team, with the exception of anesthetists. Tensions triggered by task difficulties, however, did not lower teamwork quality whereas tensions triggered by coordination problems lowered teamwork quality for team members, again with the exception of the main surgeon and the anesthetists, although the negative effect emerged as a trend (P<0.10) for both. This, again, points to the importance of potential problems in the team process–coordination [10,56]. The negative effect of tensions on teamwork is corroborated by many studies and has been found for all types of teams [57]. In turn, teamwork problems may lead to performance problems, as the team process suffers [27,28]. Tensions create negative emotions, and these have been found to impair performance in teams [58], to threaten the social integration of group members [33], and to impair learning [31].

Although not investigated in this study, there is reason to speculate that tensions in the OR can lead to negative patient outcomes and threaten patient safety [7,9,12,26,59], because problems in teamwork have been related to more errors and more complications for surgical teams [60,61].

The behaviors we studied can be regarded as indicators of an impaired “relational coordination” [10,59]. The importance of smooth coordination on a micro-level is mentioned in research in this tradition [55] as well as in some other studies (e.g. [52]). The focus of the relational coordination concept is, however, on communication patterns that characterize teams, not on the extent to which impaired coordination triggers tense, or uncivil, communication. To our knowledge, the current study is the first one to provide such results.

Our results suggest that the micro-coordination difficulties that can trigger tense communication may be routed, at least partly, in insufficiently developed shared mental models and in insufficient situation awareness, which enables team members to anticipate what will be needed next (see [55]). Tense communication evidently is not (only) a trigger of problems in the OR, but in many cases the results of problems–more specifically, of coordination problems. As the disruption often occurs prior to this tense communication, we put the term “disruptive behavior” in quotation marks in the title.

### Strength and limitations

This study assessed tense communication and their specific triggers in the OR by direct observation. The observational method allowed to disentangle influences of individual surgeons and situational aspect. The method allows a detailed look at the situations that lead to overt tense behaviors, and avoids biases related to self-report [62]. Teamwork quality was assessed immediately after each surgery. Assessing experienced tensions, their interpretation and their outcomes in the same questionnaire or interview study can lead to biases and may also be influenced by conflicts and tensions beyond specific surgeries [62].

A limitation of this study is that we included elective general surgeries in two hospitals. Generalization to other domains are thus limited. Another limitation is that verbally expressed tensions certainly do not represent all tensions in the OR. Tensions may be expressed in more subtle ways (e.g. rolling eyes) or often, may not be expressed at all. Team members often hold back and do not communicate all task, collaboration or interpersonal problems. The tensions that are expressed verbally thus are most likely only the tip of the iceberg.

Strictly speaking, causal conclusions cannot be drawn from this study. Although teamwork quality was assessed at the end of each surgery and after observation, it is possible that teamwork problems may have been among the reasons for tensions to occur; reciprocal influences are very likely [33]. Finally, our data on effects refer only to effects on perceived collaboration quality by the team members; future research should examine if tense communication has effects on the patients as well.

## Conclusions

The main lesson from this study is that tensions in the OR depend on the individual surgeon, but to a greater extent on situational triggers. Thus, explanations focusing on hierarchy, rank differences [1], power [63] or on surgeons’ personality [15] explain only part of the problem. Undeniably, surgeons have the responsibility to not overstep and to control themselves in interactions [9]; and suggestions such as conflict-management training for surgeons are often indicated [64]. Interventions helping surgeons to express dissatisfaction and critique in a firm way, but without being offensive, may be helpful, and such interventions have shown to be effective [65].

However, situational aspects that trigger tensions need to be addressed as well. Classical reasons of conflict within teams, such as disagreements about tasks, relationships, and processes [66] were either never observed or extremely rare in our study. Most tensions were triggered by aspects hindering smooth task execution or smooth coordination. For OR teams that depend on close and time-sensitive collaboration, more attention should be given to such aspects. A closer analysis of threats to smooth cooperation is needed, and the results of such analyses could feed into training that is adapted to training needs, which is this case would include micro-coordination aspects [67]. Such trainings have been shown to be effective in health care settings [68,69]; they therefore might well diminish the triggers of tensions. Furthermore, systematic team reflection on “objectives, strategies, goals, processes and outcomes” [70] could be used as a tool for improving coordination; one of its advantages is that it can be done within very short time frames [71].
